# A Secure Charging System for Electric Vehicles Based on Blockchain

**DOI:** 10.3390/s19133028

**Published:** 2019-07-09

**Authors:** MyeongHyun Kim, KiSung Park, SungJin Yu, JoonYoung Lee, YoungHo Park, Sang-Woo Lee, BoHeung Chung

**Affiliations:** 1 School of Electronics Engineering, Kyungpook National University, Daegu 41566, Korea; 2 Information Security Research Division, Electronics and Telecommunications Research Institute, Daejeon 34129, Korea

**Keywords:** smart grid, internet-of-things, blockchain, electric vehicle, secure charging system

## Abstract

Smart grids incorporating internet-of-things are emerging solutions to provide a reliable, sustainable and efficient electricity supply, and electric vehicle drivers can access efficient charging services in the smart grid. However, traditional electric vehicle charging systems are vulnerable to distributed denial of service and privileged insider attacks when the central charging server is attacked. The blockchain-based charging systems have been proposed to resolve these problems. In 2018, Huang et al. proposed the electric vehicle charging system using lightning network and smart contract. However, their system has an inefficient charging mechanism and does not guarantee security of key. We propose a secure charging system for electric vehicles based on blockchain to resolve these security flaws. Our charging system ensures the security of key, secure mutual authentication, anonymity, and perfect forward secrecy, and also provides efficient charging. We demonstrate that our proposed system provides secure mutual authentication using Burrows–Abadi–Needham logic and prevents replay and man-in-the-middle attacks using automated validation of internet security protocols and applications simulation tool. Furthermore, we compare computation and communication costs with previous schemes. Therefore, the proposed charging system efficiently applies to practical charging systems for electric vehicles.

## 1. Introduction

With widespread adoption of electric vehicles (EVs) and internet-of-things (IoT), smart grids with IoT have become promising solutions to control distributed energy and electricity generation [1]. Internet-of-things is applicable to various forms for vehicular systems, such as vehicular ad hoc networks, vehicle to grid (V2G), vehicle to vehicle (V2V), and internet of vehicle (IoV). Vehicles generally have various communication and measuring sensors, including speed, position, Bluetooth, Wi-Fi and On-board units (OBU). The sensors in vehicle collect and share data such as speed, location, identity and movements. However, an adversary can intercept, modify and reuse the sensitive data of user, and then try to obatin user’s sensitive data because it is transmitted via public network. Therefore, secure mutual authentication and key agreement must be guaranteed to provide secure communication and protect user’s privacy.

In the past decades, the numerous authentication and key agreement schemes for vehicular systems in IoT have been studied to achieve essential security requirements [2,3,4,5,6,7,8,9,10]. Although these schemes try to ensure privacy and enhance efficiency, their scheme is vulnerable to various potential attacks such as distributed denial of service and privileged insider attacks because it is based on trusted third party to provide high security level. If the trusted third party is compromised, their schemes cannot provide services. For these reasons, authenticaion and key agreement schemes without a trusted third party must be proposed to achieve integrity, confidentiality, availability and reliability, considering resource-constrained devices

The smart grid provides reliable, sustainable, stable, and efficient electricity supply. EV charging management is a particularly important issue for smart grids, and related studies have been proposed [11,12,13,14,15,16], providing EV charging for users when they want to use it. However, traditional smart grid systems provide charging services based on a third party and are vulnerable to various attacks, including distributed denial of service and privileged insider attacks. If the third party is compromised, users cannot use EV charging, and smart grid systems with IoT must also consider efficiency, because EV sensors as OBU in EV also have low power and small memory.

Several studies have proposed blockchain approaches to overcome these security weaknesses and enhance efficiency [17,18,19,20,21,22]. Blockchain [17] technology guarantees decentralization, verification, and integrity, and is applicable to various fields, including smart grids, healthcare, finance, markets, and voting. Generally, blockchains consists of data blocks, called transactions, where each transaction includes data regarding previous transactions using a hash algorithm [18]. However, early blockchain studies focused on cryptocurrency, e.g., Bitcoin and Ethereum, which have significant scalability problems. Hyperledgers [19], which do not generate cryptocurrency, have been proposed to overcome these problems and solve scalability. Huang et al. [20] proposed blockchain based EV charging management security model using smart contracts and the lightning network.

This paper demonstrates that Huang et al.’s charging system [20] has inefficient charging mechanisms such as the deposit problem, generating transaction and cost of transaction fee. Huang et al.’s system also does not guarantee security of keys. Consequently, we propose a secure charging system for electric vehicles using a hyperledger to improve security and efficiency. Our main contributions are as follows.

(1) We demonstrate security weaknesses of Huang et al.’s model, and highlight its inefficiencies.
(2) We propose a secure charging system for EV based on blockchain to solve these security weaknesses and improve efficiency.
(3) We perform informal analysis to demonstrate the proposed system is secure against various attacks, and prove that it provides secure mutual authentication using Burrows–Abadi–Needham (BAN) logic. We also perform formal security verification using the AVISPA tool and compare performances with previous schemes.
(4) We analyze the computational and communication costs compared with other related existing schemes.

### 1.1. Organization

The remainder of this paper is organized as follows. Section 2 introduces the proposed blockchain-based EV charging model. Section 3 and Section 4 review Huang et al.’s scheme and analyze its security flaws. Section 5 proposes a secure charging system for EV based on blockchain. Section 6 provides informal analysis to verify the proposed system’s security and proves that the system mutual authentication using BAN logic. We also demonstrate that the proposed system is secure against replay and man-in-the-middle attacks using the AVISPA simulation tool. Section 7 compares the proposed system’s performance with related previous schemes. Finally, Section 8 summarizes and concludes the paper.

### 1.2. Related Works

There are several authentication and key agreement schemes for wireless sensor networks to resolve privacy and security issues [23,24,25,26]. In 2011, Roman et al. [23] analyzed existing authentication and key management schemes for WSN in IoT to enhance security and performance. In 2014, Turkanovic et al. [24] first proposed an authentication and key agreement scheme based on IoT considering resource-constrained devices. However, in 2016, Amin et al. [25] showed that Turkanovic et al.’s scheme was vulnerable to smart card theft, offline identity-password guessing and user impersonation attacks, and proposed an enhanced authentication scheme to overcome these security problems. However, Lu [26] et al. pointed out that Amin et al.’s scheme did not prevent known session-specific temporary information attack and proposed improved authentication and key agreement scheme using symmetric key.

Numerous privacy protection schemes for a vehicular system in IoT ensure security and improve efficiency [2,3,4,5,6,7,8,9,10]. In 2016, Lo and Tasi [2] proposed an efficient authentication scheme for vehicular sensor networks to improve performance. Kumari et al. [3] also proposed a secure trust-extended authentication scheme for vehicular ad-hoc networks to ensure authenticity of messages. In 2017, Chin et al. [4] discussed the security vulnerabilities in the internet-of-things-based smart grid and Liu et al. [5] proposed efficient dual authentication and key agreement scheme in IoV. Mohit et al. [6] also proposed authentication protocol for smart vehicular system in IoT considering energy efficiency of sensor and Guo et al. [7] proposed secure information collection scheme for big data in large scale IoV. Zhou et al. [8] proposed an enhanced privacy-preserving authentication scheme for vehicle sensor network to overcome security weaknesses of the Kumari et al.’s scheme [3]. In 2018, Shen et al. [9] proposed privacy-preserving and lightweight key agreement scheme for V2G in social IoT to guarantee security of smart grid. In 2019, Wu et al. [10] proposed mutual authentication scheme for V2V in vehicular ad hoc network to achieve better performance and security.

Several previous studies have considered EV charging systems [11,12,13,14,15,16]. In 2013, Gan et al. [11] proposed a decentralized protocol for EV charging to improve charging efficiency. In 2016, Xu et al. [12] proposed dynamic EV scheduling using less laxity and longer remaining processing time principle. In 2010 and 2012, Lu et al. [13] and Kim et al. [14] proposed scheduling mechanisms to reduce waiting times at charging stations. In 2016, Tian et al. [15] proposed recommendation system in real-time to provide charging station information. Tang and Zhang [16] proposed a low complexity EV charging scheduling algorithm using near optimal solutions.

Many studies have guaranteed decentralization, verification, and integrity [17,19,21,22,27]. However, initial blockchain models have scalability problems. Lightning network [21] and hyperledger [19] networks have been proposed to overcome this problem and improve efficiency. Lightning networks establish trading channels outside the main blockchain to enhance network performance. Hyperledger solves scalability problem and removes the requirement for cryptocurrency transactions to enhance the performances. These systems incorporate smart contracts, a set of commitments, that improve performance and privacy without requiring a trusted third party [22,27].

Several blockchain-based EV charging management systems have been proposed to improve performance [20,28,29,30]. In 2017, Dubois et al. [28] proposed an app-based blockchain system using smart contracts to provide a charging service between EVs and charging stations without requiring a trusted third party. Knirsch et al. [29] proposed an EV charging system where the EV finds the best charging station by bidding on the blockchain. Li et al. [30] proposed a consortium blockchain for energy trading in the industrial IoT to guarantee fast and reliable energy trading using the Stackelberg game. In 2018, Huang et al. [20] proposed a blockchain-based EV charging management security model, incorporating authentication mechanisms using smart contracts and the lightning network. However, the lightning network has an inefficient charging mechanism and Huang et al.’s system does not guarantee security of keys.

## 2. System Model

This section presents a secure charging system model for EV based on blockchain and basic concept of hyperledger.

### 2.1. Hyperledger

Hyperledger [19] is an open source project proposed by the Linux Foundation in 2015. The purpose is to promote cross-industry cooperation with blockchain technology. Hyperledger focuses on enhancing blockchain performance and reliability without requiring cryptocurrency.

Hyperledger architecture comprises nine blockchain components.

1. Consensus layer: ensures agreement between transaction order and checks their validation.
2. Smart contract layer: processes transaction requests and verifies smart contracts are valid.
3. Communication layer: guarantees security for messages transmitted between nodes.
4. Data store abstraction: manages data.
5. Crypto abstraction: includes cryptographic algorithms or modules.
6. Identity service: sets up the blockchain network, manages user and system node registration, and provides authentication and authorization.
7. Policy services: provide various policies for blockchain systems.
8. APIs: provide various blockchain interfaces.
9. Interoperation: allows interoperation among blockchain instances.

Hyperledger provides scalability, confidentiality, complexity, essential security requirements, and compliance; and can also provide distributed ledgers, smart contracts, and friendly interfaces. Therefore, hyperledgers can be applied to various fields and disseminate blockchain technologies for cross-industry applications.

### 2.2. System Model

**Figure 1:**
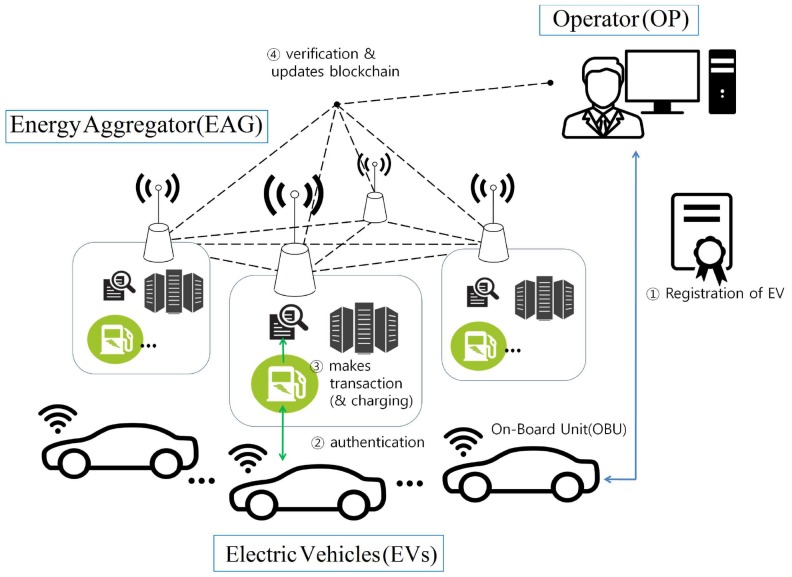
Proposed blockchain based charging system model for electric vehicle.

Figure 1 presents the proposed charging system for EV based on blockchain and our charging system comprises three entities: operator, energy aggregator (EAG), and user/EV; and incorporates four phases: initialization, registration, authentication, and charging, as follows.

1. EV registers their identity with the operator to access charging services.
2. EV and EAG authenticate each other.
3. EAG generates transactions.
4. EAG verifies the transaction is valid and records transactions on blockchain networks.

## 3. Review of Huang et al.’s Scheme

This section reviews Huang et al.’s blockchain based EV charging system [20], comprising four phases: registration, scheduling, authentication, and charging.

### 3.1. Registration Phase

**Figure 2:**
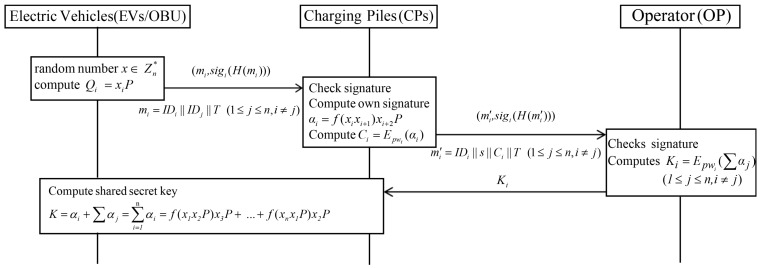
Registration phase of Huang et al.’s scheme.

Figure 2 shows how Huang scheme charging management includes EVs, charging piles (CPs) and operators (OP). Each participant is required to register in the open blockchain system. In Huang et al.’s scheme, lightning network transaction management system adopts an internet based cloud platform. The detailed steps are as follows.

**Step** **1:** EVs $\left\{V_{1},V_{2},\cdots ,V_{n}\right\}$ choose a random number $x\in Z_{n}^{*}$ and calculate $Q_{i}=x_{i}P$. The EVs request a registration in a blockchain and broadcast $m_{i}=ID_{i}||ID_{j}\left|\right|T$,(1≤j≤n,i≠j), and $\left(m_{i},sig_{i}\left(H\left(m_{i}\right)\right)\right)$.
**Step** **2:** CPs check whether the received signature is valid, and then compute signature $\alpha_{i}=f\left(x_{i}x_{i+1}\right)x_{i+2}P$ and $C_{i}=E_{pw_{i}}\left(\alpha_{i}\right)$. Then, CPs send $m_{i}=ID_{i}\left||s|\right|C_{i}\left|\right|T$ and $\left(m_{i}^{\prime },sig_{i}\left(H\left(m_{i}^{\prime }\right)\right)\right)$ to the OP in the blockchain.
**Step** **3:** OP verifies the received the signature is valid and reuses $PW_{i}$, where $PW_{i}$ is shared with each participant to decrypt $\alpha_{i}$. Subsequently OP computes $K_{i}=E_{PW_{i}}\left(\sum \alpha_{j}\right)\left(1\leq j\leq n,i\neq j\right)$ and broadcasts it in the blockchain.
**Step** **4:** After receiving key $K_{i}$ from the OP, each participant decrypts $K_{i}$ and computes session key $K=\alpha_{i}+\sum \alpha_{j}=\sum_{i=1}^{n}\alpha_{i}=f\left(x_{1}x_{2}P\right)x_{3}P+\cdots +f\left(x_{n}x_{1}P\right)x_{2}P$. When a request is recorded in the blockchain, it is checked by all CPs.

### 3.2. Scheduling Phase

**Figure 3:**
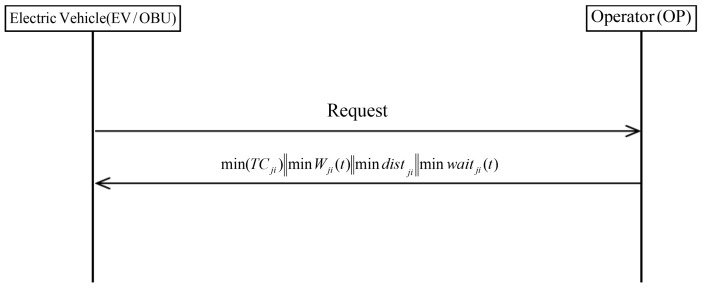
Scheduling phase of Huang et al.’s scheme.

Figure 3 shows the Huang scheme includes four scheduling strategies to provide charging services and improve charging efficiency: minimum time, minimum waiting time, minimum total cost, and shortest path.

### 3.3. Authentication Phase

**Figure 4:**
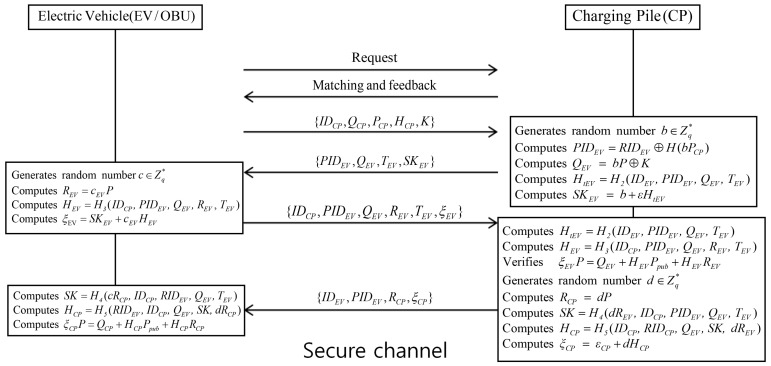
Authentication phase of Huang et al.’s scheme.

Figure 4 shows that EV and CP perform point-to-point (P2P) transactions under the Huang scheme, followed with mutual authentication to improve trading flexibility. Depending on scheduling strategies, the EV receives the recommendation from the OP, then EV and the selected CP authenticate each other, with mutual authentication performed between EV and CP when the EV arrives at the CP. The detailed authentication steps are as follows.

**Step** **1:** EV sends identity $ID_{EV}$ to the CP, and then the CP checks whether it is valid through the blockchain networks. If it is valid, the CP returns charging request to the EV.
**Step** **2:** After receiving the charging request from the CP, the EV sends $\left\{Q_{CP},P_{CP},ID_{CP},H_{CP},K\right\}$ to the CP.
**Step** **3:** CP selects a random number $b\in Z_{q}^{*}$, current timestamp $T_{i}$, and calculates $PID_{EV}=RID_{EV}⊕H_{1}\left(bP_{CP}\right)$, $Q_{EV}=bP⊕K$, $H_{tEV}=H_{2}(ID_{EV}$, $PID_{EV},Q_{EV},T_{EV})$ and $SK_{EV}=b+\epsilon H_{tEV}$. Then, the CP sends $\{PID_{EV},Q_{EV}$, $T_{EV},SK_{EV}\}$ to the EV.
**Step** **4:** EV selects a random number $c\in Z_{q}^{*}$, calculates $R_{EV}$=$c_{EV}P$, $H_{EV}$$=H_{3}(ID_{CP}$, $PID_{EV},Q_{EV},R_{EV},T_{EV})$ and $\xi_{EV}=SK_{EV}+c_{EV}H_{EV}$, and sends $\{ID_{CP}$, $PID_{EV},Q_{EV},R_{EV},T_{EV},\xi_{EV}\}$ to the CP via the secure channel.
**Step** **5:** After receiving the message from the EV, the CP computes $H_{tEV}$=$H_{2}$$\left(ID_{EV},PID_{EV},Q_{EV},T_{EV}\right)$ and $H_{EV}=H_{3}\left(ID_{CP},PID_{EV},Q_{EV},R_{EV},T_{EV}\right)$, and checks the received signature. If it is not valid, the CP aborts the authentication phase. Otherwise, the CP selects a random number $d\in Z_{q}^{*}$ and computes $R_{CP}=dP$, $SK=H_{4}\left(dR_{EV},ID_{CP},PID_{EV},Q_{EV},T_{EV}\right)$, $H_{CP}=H_{5}\left(ID_{CP},RID_{CP},Q_{EV},SK,dR_{EV}\right)$, and $\xi_{CP}=\epsilon_{CP}+dH_{CP}$. Finally, the CP sends $\left\{ID_{EV},PID_{EV},R_{CP},\xi_{CP}\right\}$ to the EV.
**Step** **6:** After receiving the message from the CP, the EV computes $SK=H_{4}(cR_{CP},ID_{CP}$, $RID_{EV},Q_{EV},T_{EV})$,$H_{CP}=H_{5}(RID_{EV}$, and $ID_{CP},Q_{EV},SK,dR_{CP})$. The CP checks the received signature. If valid, the EV and CP achieve mutual authentication. Otherwise, the EV aborts mutual authentication. After mutual authentication, the session key SK is used to encrypt messages to ensure secure communications.

### 3.4. Charging Phase

**Figure 5:**
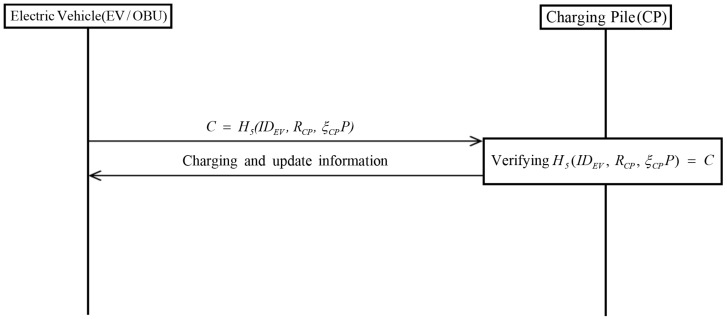
Charging phase of Huang et al.’s scheme.

Figure 5 shows the Huang scheme charging phase. After completing authentication, the EV performs charging and updates the transactions. The commitment is recorded in the blockchain. The detailed steps are as follows.

**Step** **1:** EV calculates commitment $C=H_{5}\left(ID_{EV},R_{CP},\xi_{CP}P\right)$ including EV’s identity, random parameter, and signature.
**Step** **2:** CP verifies whether commitment $C=H_{5}\left(ID_{EV},R_{CP}\xi_{CP}P\right)$ and current time stamps are valid. If valid, the CP starts charging for EV. Otherwise, this phase is aborted.

## 4. Problems of Huang et al.’s Scheme

This section discusses problems of Huang et al.’s scheme, such as deposit problem, inefficient transaction generation mechanism, and transaction cost.

### 4.1. Deposit Problem

In the scheduling phase of Huang et al.’s scheme, the OP recommends an efficient charging station using four strategies. However, the system has a deposit problem where EV needs to establish many payment channels for charging. In lightning networks, a deposit is used to establish a payment channel between two participants. However, an EV is mobile and cannot efficiently choose optimal charging stations because a payment channel must be established with each new charging station. Therefore, an EV unnecessarily spends many deposits, i.e., the mechanism is inefficient.

### 4.2. Inefficient Mechanism for Generating Transaction

The lightning network records a transaction in the blockchain when opening and closing a payment channel. When charging is completed at a charging station, the commitment transaction is generated and recorded in the blockchain. However, an EV already makes a transaction in the registration phase to allows access to the service, hence, two transactions are generated for one trade, i.e., an inefficient charging mechanism.

### 4.3. Cost of Transaction Fee

The Huang et al.’s scheme charges a transaction fee when a payment channel is opened or closed. However, after completing charging, an EV needs to re-register with the OP to use the service, hence re-opening the payment channel and incurring a transaction fee. Therefore, the Huang et al.’s scheme results in high transaction fees.

### 4.4. Security of Key

Huang et al. claimed their scheme provided known key security because the shared secret key *K* included unique random numbers for each participant. However, an adversary can steal the EV on-board unit (OBU) [31] and extract sensitive data stored in its memory using the power analysis attack [32,33]. Consequently, an adversary can obtain shared secret keys stored in the OBU, avoiding known key security.

## 5. Proposed Charging System for EV Based on Blockchain

This section proposes a blockchain based EV charging system that addresses the identified problems. Table 1 details the notation used for the proposed phases: initialization, registration, authentication, and charging.

### 5.1. Initialization Phase

The operator conducts system initialization to set up the networks and EAG is registered in network. The details initialization process is as follows.

**Step** **1:** OP selects a base point *G* on the elliptic curve $E_{p}$ with order *n*, where *n* is a large prime number.
**Step** **2:** EAG generates public key, private key, and random number $b_{1}$.
**Step** **3:** OP defines network configuration including channel members and policies, records it in a blockchain, and shares it with system participants.

### 5.2. Registration Phase

**Figure 6:**
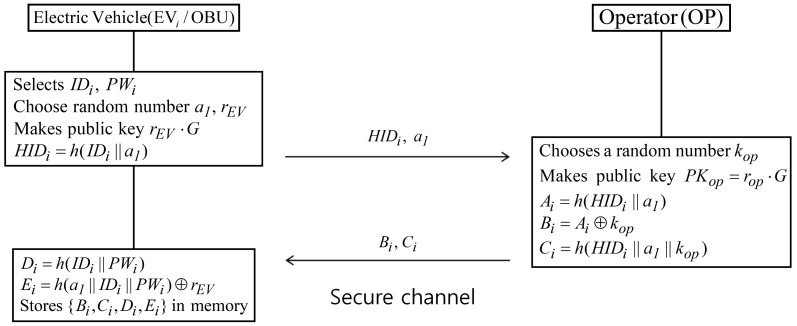
Registration phase of proposed scheme

When it wants to access the charging system, EV${}_{i}$ generates its identity, password, public/private key pair, and then receives a random number from OP. Figure 6 shows the registration phase, with detailed steps as follows.

**Step** **1:** EV${}_{i}$ selects their identity $ID_{i}$, password $PW_{i}$, generates random numbers $a_{1}$ and $r_{EV}$, calculates a public key $r_{EV}\cdot G$ and $HID_{i}=h(ID_{i}\left|\right|a_{1})$, and then sends $HID_{i}$ and $a_{1}$ to OP through a secure channel.
**Step** **2:** OP chooses a random number $k_{op}$ and calculates public key $PK_{op}=r_{op}\cdot G$, $A_{i}=h(HID_{i}\left|\right|a_{1})$, $B_{i}=A_{i}⊕k_{op}$, and $C_{i}=h(HID_{i}\left|\right|a_{1}\left|\right|k_{op})$. Finally, OP sends $\{B_{i}$, $C_{i}\}$ to EV${}_{i}$, modifies network configurations, and stores details in the blockchain.
**Step** **3:** EV${}_{i}$ computes $D_{i}=h(ID_{i}||PW_{i})⊕a_{i}$, $E_{i}=h(a_{1}||ID_{i}||PW_{i})⊕r_{EV}$; and stores $\{B_{i}$, $C_{i}$, $D_{i}$, and $E_{i}\}$ in memory.

### 5.3. Authentication Phase

**Figure 7:**
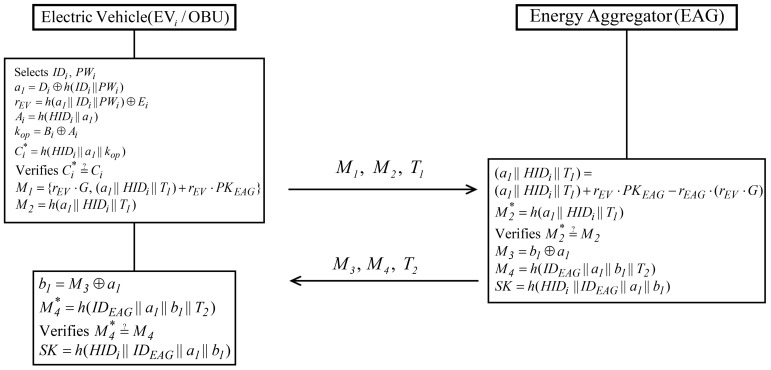
Authentication phase of proposed scheme.

When EV${}_{i}$ wants to use the charging service, EV${}_{i}$ and EAG must authenticate each other, and then generate a common session key. Figure 7 shows the authentication phase with detailed steps as follows

**Step** **1:** EV${}_{i}$ inputs identity $ID_{i}$ and password $PW_{i}$; and calculates $a_{1}=D_{i}⊕h(ID_{i}||PW_{i})$, $r_{EV}=h(a_{1}||ID_{i}||PW_{i})⊕E_{i}$, $A_{i}=h(HID_{i}\left|\right|a_{i})$, $k_{op}=B_{i}⊕A_{i}$, and $C_{i}^{*}=h(HID_{i}\left|\right|a_{1}\left|\right|k_{op})$. Then, EV${}_{i}$ checks whether $C_{i}^{*}\overset{?}{=}C_{i}$. If valid, EV${}_{i}$ computes $M_{1}=\{r_{EV}\cdot G,(a_{1}||HID_{i}\left|\right|T_{i})+r_{EV}\cdot PK_{EAG}\}$, and $M_{2}=h(a_{1}||HID_{i}\left|\right|T_{1})$; and sends $\{M_{1}$, $M_{2}$, $T_{1}\}$ to EAG.
**Step** **2:** After receiving $\{M_{1}$, $M_{2}$, $T_{1}\}$ from EV${}_{i}$, EAG calculates $(a_{1}||HID_{i}\left|\right|T_{1})=(a_{1}||HID_{i}\left|\right|T_{1})+r_{EV}\cdot PK_{EAG}-r_{EAG}\cdot \left(r_{EV}\cdot G\right)$ using the private key $r_{EAG}$. Then EAG computes $M_{2}^{*}=h(a_{1}||HID_{i}\left|\right|T_{1})$ and verifies $M_{2}^{*}\overset{?}{=}M_{2}$. If valid, EAG authenticates EV${}_{i}$ and calculates $M_{3}=b_{1}⊕a_{1}$, $M_{4}=h(ID_{EAG}\left|\right|a_{1}\left|\right|b_{1}\left|\right|T_{2})$, and session key $SK=h(HID_{i}||ID_{EAG}||a_{1}||b_{1})$. Finally, EAG sends $\{M_{3}$, $M_{4}$, $T_{2}\}$ to EV${}_{i}$.
**Step** **3:** When EV${}_{i}$ receives $\{M_{3}$, $M_{4}$, $T_{2}\}$ from EAG, it computes $b_{1}=M_{3}⊕a_{1}$ and $M_{4}^{*}=h(IE_{EAG}\left|\right|a_{1}\left|\right|b_{1}\left|\right|T_{2})$, and verifies $M_{4}^{*}\overset{?}{=}M_{4}$. If valid, mutual authentication between EV${}_{i}$ and EAG has been accomplished. EV${}_{i}$ calculates a shared session key, $SK=h(HID_{i}||ID_{EAG}||a_{1}||b_{1})$.

### 5.4. Charging Phase

**Figure 8:**
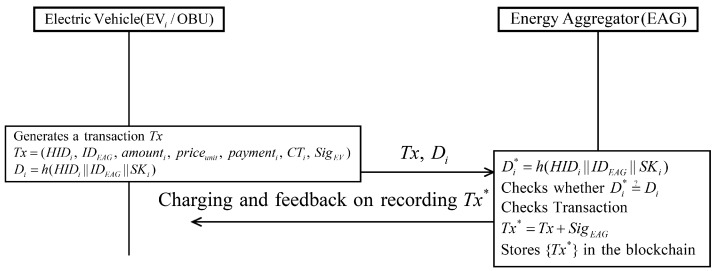
Charging phase of proposed scheme.

Charging commences after successfully completing authentication. Figure 8 shows the charging phase with detailed steps as follows.

**Step** **1:** EV${}_{i}$ generates a transaction $T_{x}$ including $HID_{i}$; $ID_{EAG}$; charging records, prices, charging time $CT_{i}$, signature of EV${}_{i}$ and information of payment, and EV${}_{i}$ computes $D_{i}=h(HID_{i}||ID_{EAG}||SK_{i})$. After that, EV${}_{i}$ sends $\left\{T_{x},D_{i}\right\}$ to the EAG.
**Step** **2:** After receiving the message from EV${}_{i}$, EAG checks whether $D_{i}^{*}\overset{?}{=}D_{i}$. If it is correct, EAG checks whether the transaction information $T_{x}$ is correct. Then, EAG adds the own signature to the transaction. Finally, the charging is started and EAG records the transaction $T_{x}$ on the blockchain.

## 6. Security Analysis

This section demonstrates that the proposed system is secure against various attacks using informal analysis and proves the system provides secure mutual authentication using BAN logic [34], a widely accepted formal security analysis. We also show it is secure against replay and man-in-the-middle attacks using automated validation of internet security protocols and applications (AVISPA) [35] which is a formal security verification tool.

### 6.1. Informal Security Analysis

We perform informal security analysis to evaluate the proposed system security, and show the system can resist impersonation, replay, perfect forward secrecy, and session key disclosure attacks; and also provides secure mutual authentication and anonymity.

#### 6.1.1. Impersonation Attack

If adversary EV${}_{A}$ attempts to impersonate a legitimate user EV${}_{i}$, EV${}_{A}$ must successfully generate a request message $M_{2}=h(a_{1}||HID_{i}\left|\right|T_{1})$. However, EV${}_{A}$ cannot calculate the request message because they cannot obtain EV${}_{i}$’s random number $a_{1}$, and real identity $ID_{i}$. EV${}_{A}$ also cannot obtain EV${}_{i}$’s sensitive information because transmitted message $M_{1}$ is encrypted by EV${}_{i}$. Therefore, the proposed protocol prevents impersonation attack.

#### 6.1.2. Session Key Disclosure Attack

In the proposed system, the adversary EV${}_{A}$ cannot generate a valid session key $h(HID_{i}||ID_{EAG}||a_{1}||b_{1})$ because EV${}_{A}$ cannot obtain EV${}_{i}$’s real identity $ID_{i}$; or random numbers, $a_{1}$ and $b_{1}$, generated by EAG. EV${}_{A}$ also cannot decrypt $M_{1}$ without EV${}_{i}$’s private key $r_{EV}$. Therefore, the proposed protocol can resist session key disclosure attack.

#### 6.1.3. Perfect Forward Secrecy

Even if an EV${}_{i}$ long-term private parameter of EV${}_{i}$ is compromised, EV${}_{A}$ does not obtain the previous session key. Suppose the long-term private parameter $a_{1}$ is leaked. EV${}_{A}$ cannot obtain $ID_{i}$ because they cannot decrypt $M_{1}$ without the correct private key. Therefore, the proposed protocol guarantees perfect forward secrecy.

#### 6.1.4. Replay Attack

Suppose EV${}_{A}$ attempted a replay attack using previous transmitted messages. However, EV${}_{A}$ cannot reuse previous messages, because all transmitted messages include timestamps, and EV${}_{i}$ and EAG check all transmitted messages including the timestamps are correct. Therefore, the proposed protocol prevents replay attack.

#### 6.1.5. Mutual Authentication

Section 6.1.1 and Section 6.1.2 demonstrate that EV${}_{A}$ cannot impersonate EV${}_{i}$ and obtain the session key. All transmitted parameters are checked by EV${}_{i}$ and EV${}_{A}$. When EAG receives the request message $\left\{M_{1},M_{2},T_{1}\right\}$ from EV${}_{i}$, EAG decrypts $M_{1}$ using its own private key, and verifies $M_{2}$. If valid, EAG authenticates EV${}_{i}$. When EV${}_{i}$ receives response $\left\{M_{3},M_{4},T_{2}\right\}$ from EAG, it computes $b_{1}=M_{3}⊕h(HID_{i}\left|\right|a_{1})$ and $M_{4}^{*}=h(ID_{EAG}\left|\right|a_{1}\left|\right|b_{1}\left|\right|T_{2})$, and then verifies $M_{4}^{*}=h(ID_{EAG}\left|\right|a_{1}\left|\right|b_{1}\left|\right|T_{2})$. If valid, EV${}_{i}$ authenticates EAG. Therefore, the proposed system provides secure mutual authentication because EV${}_{i}$ and EAG successfully authenticate each other using private keys.

#### 6.1.6. Anonymity

Suppose EV${}_{A}$ intercepts all previous transmitted messages to attempt to obtain EV${}_{i}$’s real identity. However, all EV${}_{i}$ parameters, including $ID_{i}$, are masked by hash or encryption. Therefore, the proposed protocol ensures anonymity.

### 6.2. Security Analysis Using BAN Logic

We perform a security analysis using BAN logic to demonstrate the proposed system provides secure mutual authentication between EV and EAG. Table 2 introduces BAN logic notations and defines the goals, rules, idealized forms, and assumptions to perform BAN logic analysis.

#### BAN Logic Rules

The BAN logic rules are as follows.

**1.**  Message meaning rule:
  $$\frac{A\;|\equiv A\overset{K}{↔}B,\;A⊲{\left\{X\right\}}_{K}}{A\;\left|\equiv B\;\right|∼X}$$
**2.**  Nonce verification rule:
  $$\frac{A\;\left|\equiv \#(X),\;A\;\right|\equiv B\;|∼X}{A\;\left|\equiv B\;\right|\equiv X}$$
**3.**  Jurisdiction rule:
  $$\frac{A\;\left|\equiv B\;\right|⟹X,\;A\;\left|\equiv B\;\right|\equiv X}{A\;|\equiv X}$$
**4.**  Freshness rule:
  $$\frac{A\;|\equiv \#(X)}{A\;|\equiv \#\left(X,Y\right)}$$
**5.**  Belief rule:
  $$\frac{A\;|\equiv \left(X,Y\right)}{A\;|\equiv X.}$$

### 6.3. Goals

We define the following security goals to prove the proposed system ensures secure mutual authentication.

**Goal** **1:** $EV|\equiv (EV\overset{SK}{⟷}EAG)$
**Goal** **2:** $EV|\equiv EAG|\equiv (EV\overset{SK}{⟷}EAG)$
**Goal** **3:** $EAG|\equiv (EV\overset{SK}{⟷}EAG)$
**Goal** **4:** $EAG|\equiv EV|\equiv (EV\overset{SK}{⟷}EAG)$

#### 6.3.1. Idealized Forms

The idealized forms are given below.

$Msg_{1}$:
EV→EAG: $\left(a_{1},HID_{i},T_{1}\right)\overset{PK_{EAG}}{{}_{⟶EAG}}$
$Msg_{2}$:
EAG→EV: ${\left(b_{1},ID_{EAG},T_{2}\right)}_{a_{1}}$

#### 6.3.2. Assumptions

We define the following initial assumptions for the BAN logic proof.

$A_{1}$:
$EAG|\equiv \#(T_{1})$
$A_{2}$:
$EV|\equiv \#(T_{2})$
$A_{3}$:
$EV|\equiv (EAG\overset{a_{1}}{⟷}EV)$
$A_{4}$:
$EAG|\equiv \#(PK_{EAG})$
$A_{5}$:
$EAG|\equiv \#(a_{1})$
$A_{6}$:
$EV|\equiv \#(b_{1})$
$A_{7}$:
$EV|\equiv EAG\Rightarrow (EV\overset{SK}{⟷}EAG)$
$A_{8}$: $EAG|\equiv EV\Rightarrow (EV\overset{SK}{⟷}EAG)$

#### 6.3.3. Proof using BAN Logic

The detailed steps of the BAN logic proof are as follows.

**Step** **1:** From $Msg_{1}$,
  $$S_{1}:EAG⊲\left(a_{1},HID_{i},T_{1}\right)\overset{PK_{EAG}}{{}_{⟶EAG}}$$
**Step** **2:** From the message meaning rule with $S_{1}$ and $A_{4}$,
  $$S_{2}:EAG|\equiv EV∼\left(a_{1},HID_{i},T_{1}\right)$$
**Step** **3:** Using the freshness rule with $A_{1}$,
  $$S_{3}:EAG|\equiv \#\left(a_{1},HID_{i},T_{1}\right)$$
**Step** **4:** From the nonce verification rule with $S_{2}$ and $S_{3}$,
  $$S_{4}:EAG|\equiv EV|\equiv \left(a_{1},HID_{i},T_{1}\right)$$
**Step** **5:** Since the session key $SK=h(HID_{i}||ID_{EAG}||a_{1}||b_{1})$, from $S_{4}$ and $A_{5}$,
  $$S_{5}:EAG|\equiv EV|\equiv \left(EV\overset{SK}{⟷}EAG\right)\;\;\left(\mathrm{Goal}4\right)$$
**Step** **6:** From the jurisdiction rule with $S_{6}$ and $A_{8}$,
  $$S_{6}:EAG|\equiv \left(EV\overset{SK}{⟷}EAG\right)\;\;\left(\mathrm{Goal}3\right)$$
**Step** **7:** From the $Msg_{2}$,
  $$S_{7}:EV⊲{\left(b_{1},ID_{EAG},T_{2}\right)}_{a_{1}}$$
**Step** **8:** Using the message meaning rule with $S_{8}$ and $A_{3}$,
  $$S_{8}:EV|\equiv EAG∼{\left(b_{1},ID_{EAG},T_{2}\right)}_{a_{1}}$$
**Step** **9:** From the freshness rule with $A_{2}$,
  $$S_{9}:EV|\equiv \#{\left(b_{1},ID_{EAG},T_{2}\right)}_{a_{1}}$$
**Step** **10:** From the nonce verification rule with $S_{9}$ and $S_{10}$,
  $$S_{10}:EV|\equiv EAG|\equiv {\left(b_{1},ID_{EAG},T_{2}\right)}_{a_{1}}$$
**Step** **11:** Since the session key $SK=h(HID_{i}||ID_{EAG}||a_{1}||b_{1})$, from $S_{11}$ and $A_{6}$,
  $$S_{11}:EV|\equiv EAG|\equiv \left(EV\overset{SK}{⟷}EAG\right)\;\;\left(\mathrm{Goal}2\right)$$
**Step** **12:** From the jurisdiction rule with $S_{13}$ and $A_{7}$,
  $$S_{12}:EV|\equiv \left(EV\overset{SK}{⟷}EAG\right)\;\;\left(\mathrm{Goal}1\right)$$

Therefore, the proposed protocol achieves secure mutual authentication between EV and EAG.

### 6.4. Formal Security AVISPA Tool for Formal Security Verification

To prove the proposed system is secure against replay and man-in-the-middle attacks, we performed formal security analysis using AVISPA [35], a widely accepted formal security verification tool to check systems or protocols can resist replay and man-in-the-middle attacks [36,37,38,39].

The AVISPA module was written in a high level protocol specification language (HLPSL) [40] and consists of four backends: Tree Automate-based Protocol Analyser (TA4SP), SAT-based Model-Checker (SATMC), CL-based Attack Searcher(CL-AtSe) [41] and On-the-Fly Model-Checker(OFMC) [42]. Detailed AVISPA and HLPSL are presented in [35,40].

#### 6.4.1. HLPSL Specification of AVISPA Simulation

**Figure 9:**
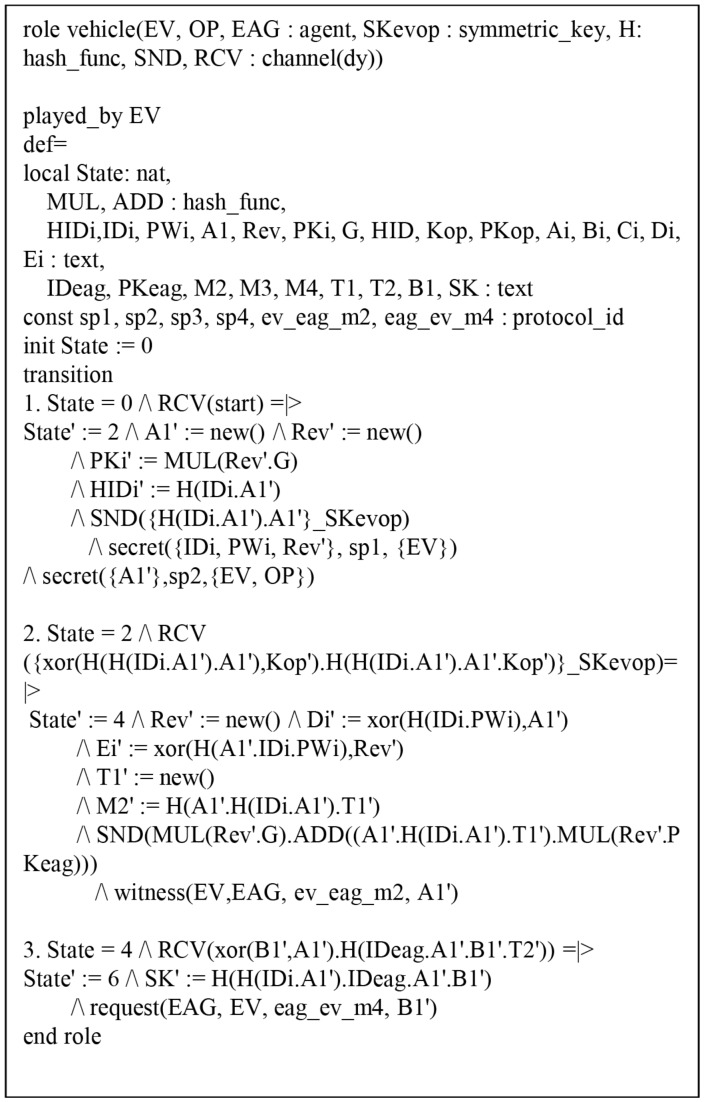
Specification of the electronic vehicle.

**Figure 10:**
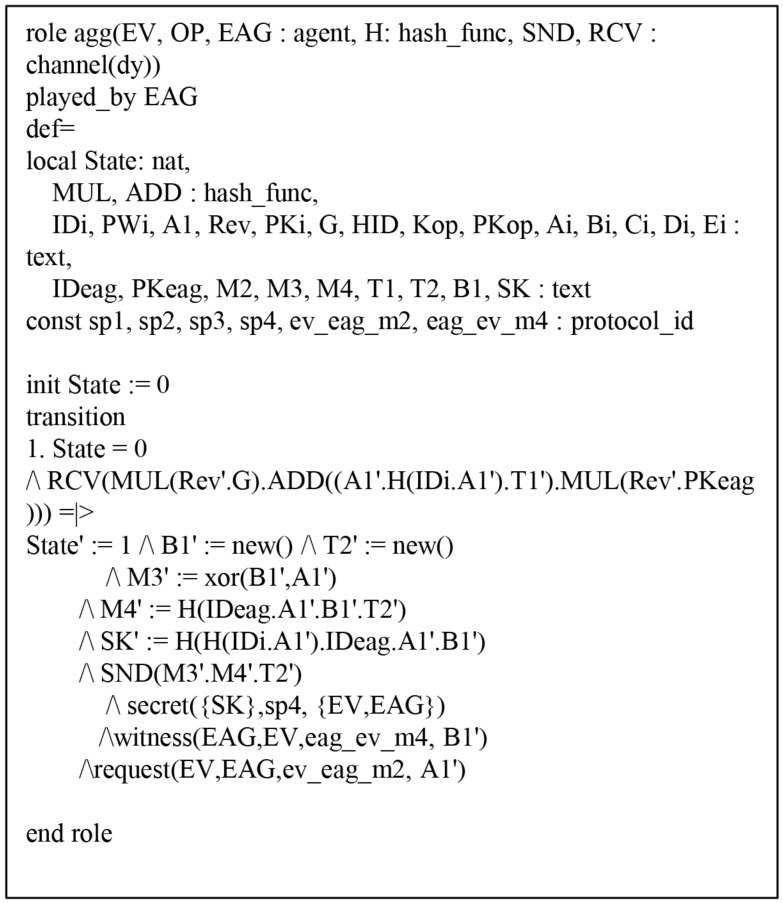
Specification of the energy aggregator.

**Figure 11:**
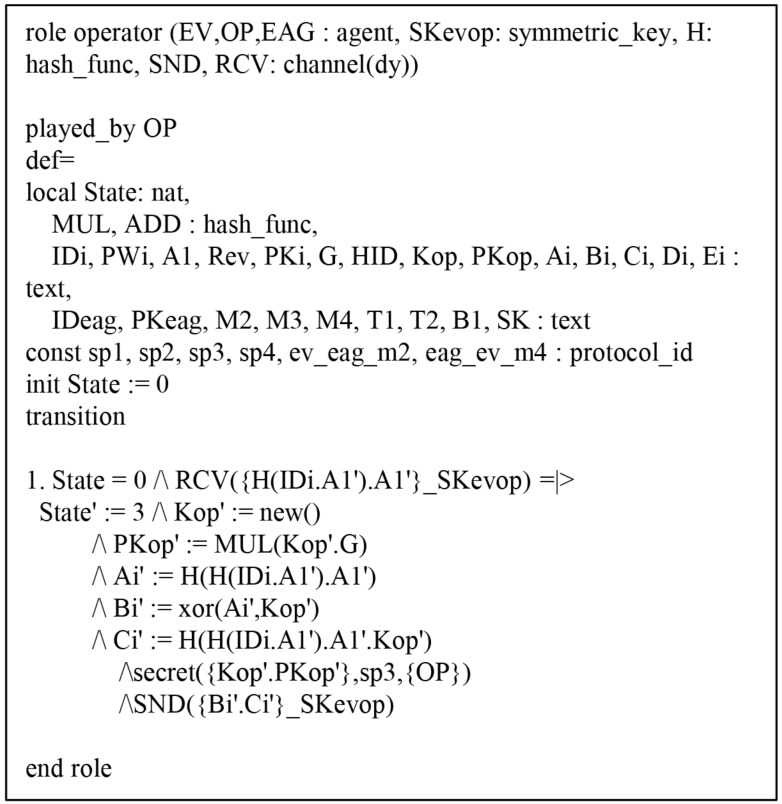
Specification of the operator.

**Figure 12:**
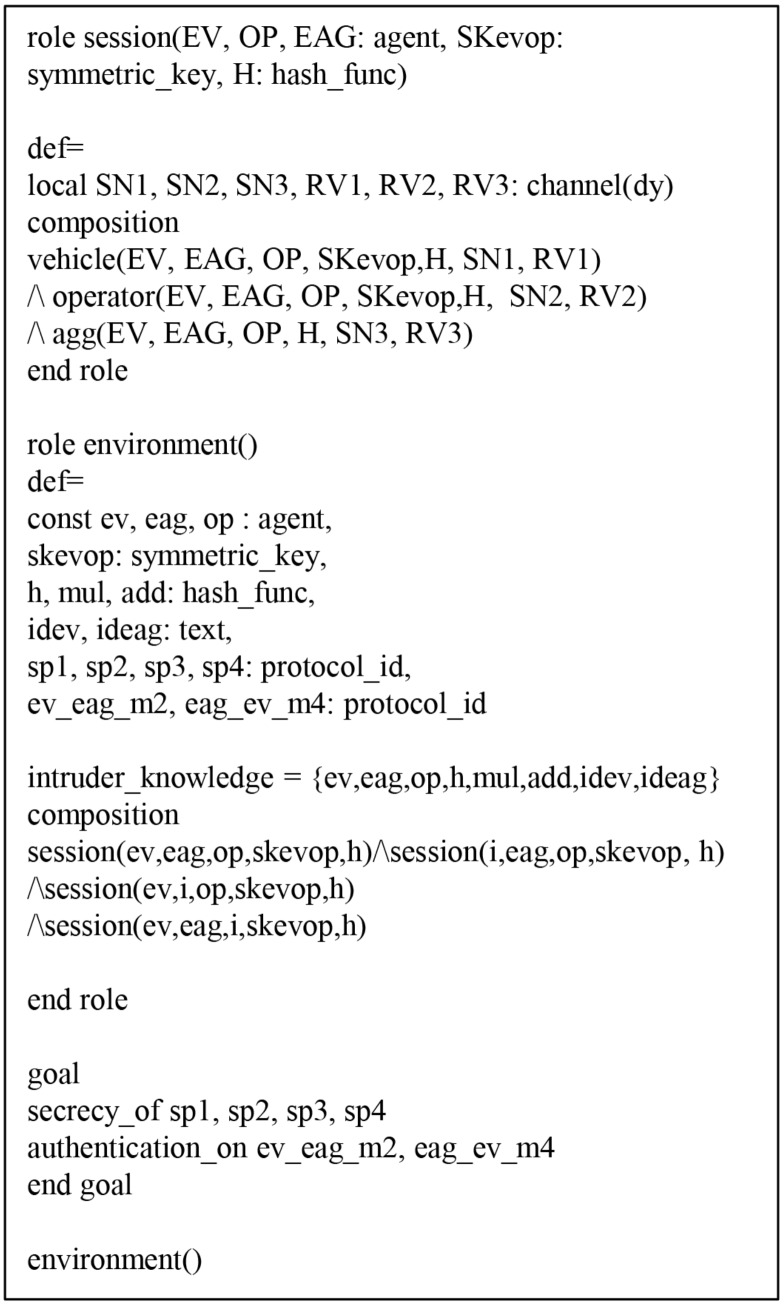
Specification of the session.

The HLPSL consists of three components: *role*, *session*, and *environment*, where *role* denotes entity, *session* includes system parameters, and *environment* includes intruder knowledge, security goal and authentication goal. Figure 9, Figure 10 and Figure 11 show HLPSL specifications for EV, EAG, and OP, respectively. Figure 12 shows *session* and *environment* roles.

Figure 9 shows the *role* for EV. In state 0, EV receives the start request and performs registration. EV sends the registration request $\left\{HID_{i},a_{1}\right\}$ to OP via a secure channel, updates the state value to 2, and then checks whether the entity is a legitimate user using the *secret* function.

In state 4, EV receives response $\left\{B_{i},C_{i}\right\}$ from OP through a secure channel and sends the login request $\left\{M_{1},M_{2},T_{1}\right\}$ to EAG via an open channel. EV also declares *witness*$\left(\mathrm{EV},\mathrm{EAG},ev\_eag\_m2,A1^{\prime }\right)$ to prove that $a_{1}$ is a weakness authentication factor. EV receives the response $\left\{M_{3},M_{4},T_{2}\right\}$ from OP, and then calculates the session key and updates the state value to 6.

In state 6, EV declares *request*$\left(\mathrm{EAG},\mathrm{EV},egg_{e}v_{m}4,B1^{\prime }\right)$ to authenticate each other. The HLPSL specifications for EAG and OP roles are described similarly (see Figure 10 and Figure 11).

#### 6.4.2. AVISPA Verification Results

**Figure 13:**
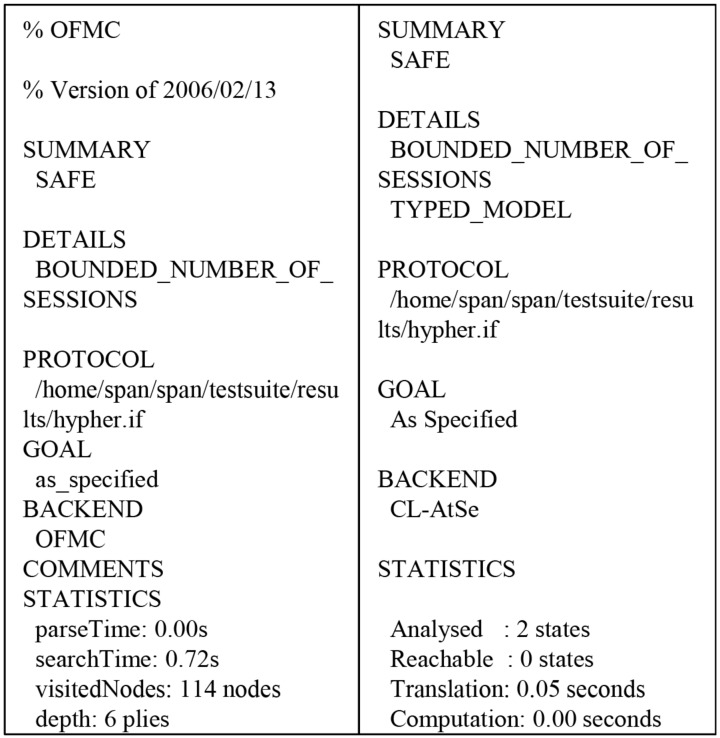
AVISPA simulation results.

We used AVISPA with security protocol animator (SAPN) [43] to evaluate whether the proposed system was secure against replay and man-in-the-middle attacks. HLPSL is translated to intermediate format (IF) and the simulation results are presented in output format (OF). OFMC and CL-AtSe check whether our proposed system prevent replay and man-in-the-middle attacks. Figure 13 shows that OFMC backend visited 114 nodes with 0.72 s search time, and CL-AtSe backend analyzed the 2 states with 0.05 s translation time.

## 7. Performance Analysis

**Figure 14:**
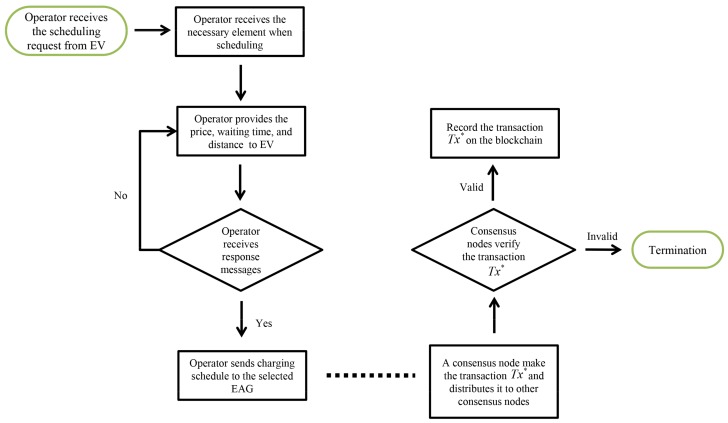
Actual data workflow of network.

**Figure 15:**
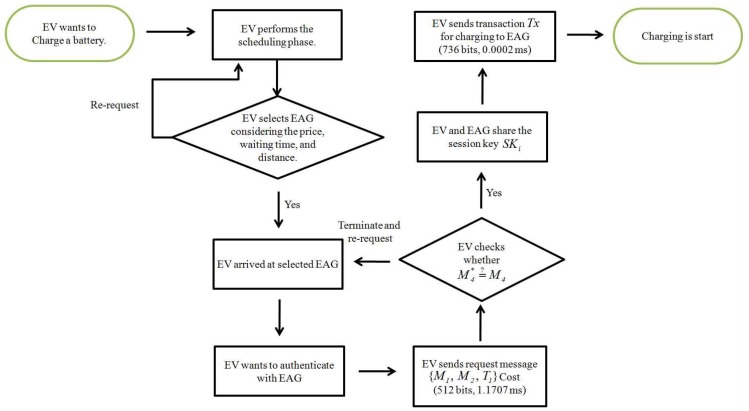
Actual data workflow of EV.

**Figure 16:**
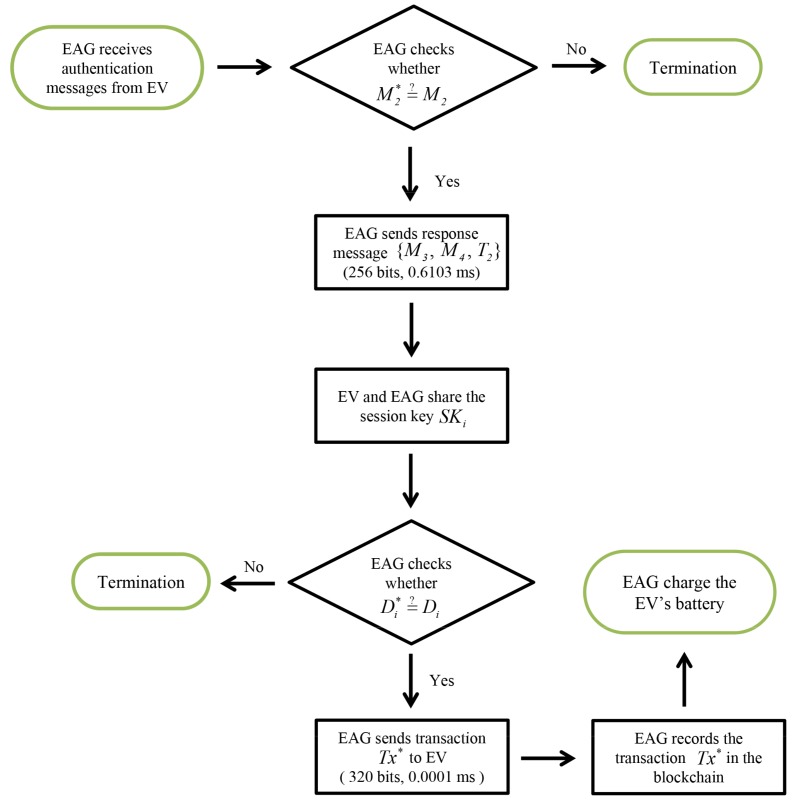
Actual data workflow of EAG.

This section compares the proposed system computation and communication costs with related schemes [20,44,45]. Figure 14, Figure 15 and Figure 16 present the actual data workflow transmitted on our system to help understand the results of performance analysis. In Figure 14, operator provides the data for scheduling to EV and consensus nodes on blockchain verifty the validity of transactions.

### 7.1. Computation Cost

We compare computation overheads for the proposed system with related schemes [20,44,45], using the parameters from [46,47].

- Bilinear pairing, $T_{bp}=4.211$ ms.
- Scalar multiplication with bilinear pairing,$T_{sm-bp}=1.709$ ms.
- Point addition with bilinear pairing, $T_{pa-bp}=0.007$ ms.
- Scalar multiplication with elliptic curve cryptography, $T_{sm-ecc}=0.442$ ms.
- Point addition with elliptic curve cryptography, $T_{pa-ecc}=0.0018$ ms.
- Encryption with elliptic curve cryptography, $T_{enc-ecc}=1.17$ ms.
- Decryption with elliptic curve cryptography, $T_{dec-ecc}=0.61$ ms.
- Hash, $T_{h}=0.0001$ ms.
- Map-to-point,$T_{mtp}=4.302$ ms.

Table 3 compares computation costs for the considered schemes. In Figure 15, the EV’s computation costs of our system are (1.1707 + 0.0002 = 1.1709 ms), including $T_{enc-ecc}$ and $9T_{h}$. In Figure 16, the EAG’s computation costs of our system are (0.6103 + 0.0001 = 0.6104 ms), including $T_{dec-ecc}$ and $4T_{h}$.

In Huang et al.’s system [20], EV’s total computation costs are (0.4459 + 0.0001 = 0.4460 ms), including $T_{sm-ecc}$, $2T_{pa-ecc}$ and $4T_{h}$. EAG’s total computation cost is 0.8882 ms, including $2T_{sm-ecc}$, $2T_{pa-ecc}$ and $7T_{h}$.

The total cost for the proposed and Huang et al.’s scheme = 1.7811 and 1.3343 ms, respectively. Although the proposed protocol computation cost of somewhat higher than the Huang et al.’s scheme, it provides better efficiency and security than other related schemes.

### 7.2. Communication Cost

We compare communication overheads for the proposed system with related schemes [20,44,45]. We assume timestamp, random number and identity are 32, 64, and 128 bits, respectively [48,49,50]; and elliptic curve cryptography encryption and hash function are 320 and 160 bits, respectively. For the proposed system, we assume group *G* is generated by *P* with order *q* on elliptic curve cryptography $y^{2}=x^{3}+ax+b$ mod *p*, where *p* and *q* are 160 bits prime numbers. Similarly, $G_{1}$, $G_{2}$, and *G* are 1024, 160, and 320 bits, respectively. In Figure 15, the EV’s communication costs of our system are (512 + 736 = 1248 bits), including charging request messages $\left\{M_{1},M_{2},T_{1}\right\}$ and transaction $T_{x}$. In Figure 16, the EAG’s communication costs of our system are (256 + 320 = 576 bits), including response messages $\left\{M_{3},M_{4},T_{2}\right\}$ and transaction $T_{x}^{*}$.

In Huang et al.’s system [20], EV’s total communication costs are (2368 + 160 = 2528 bits), including charging requset messages $\left\{ID_{CP},Q_{CP},P_{CP},K\right\}$, response messages $\left\{ID_{CP},PID_{EV},Q_{EV},R_{EV},T_{EV},\zeta_{EV}\right\}$ and charging request messages $H_{5}\left(ID_{EV},R_{CP},\zeta_{CP},P\right)$. EAG’s total communication costs are 1760 bits, including response messages $\left\{PID_{EV},Q_{EV},T_{EV},SK_{EV}\right\}$ and $\left\{ID_{EV},PID_{EV},R_{CP},\zeta_{CP}\right\}$.

Table 4 shows communication cost results for all considered schemes. More particularly, the communication cost is efficient compared with other schemes in view of EVs. It is a very important advantage because EV is equipped with resourse-constrained devices as sensors. Thus, the proposed system provides more efficient communication than all other considered schemes.

## 8. Conclusions

With the rapid development of the IoT and embedded technologies, drivers can access various services. However, these services are vulnerable to potential attacks such as replay, impersonation and session key disclosure attacks because they are provided through public channels. Many traditional cryptographic algorithms such as RSA also are suitable to vehicular networks because a vehicle is equipped with resource-constrained sensors. Therefore, secure mutual authentication and key agreement are very important security requirements to guarantee privacy of users, considering the resource-constrained sensors.

This paper demonstrated that Huang et al.’s scheme does not provide high efficiency and security of keys, and has various authentication flaws, including excess deposits, inefficient transaction generation, and excess transaction fees. We proposed a secure charging system for electric vehicles based on blockchain to address these weaknesses, providing high efficiency, anonymity, perfect forward secrecy, and secure mutual authentication. We demonstrated that the proposed system prevents impersonation, session key disclosure, and replay attacks, proved secure mutual authentication between EV and EAG using BAN logic, and confirmed resistance to replay and man-in-the-middle attacks using AVISPA. We compared computation and communication costs with related schemes, and showed that the proposed scheme was superior to all considered schemes. Thus, the proposed system could be applied to IoT-based practical EV charging systems for resource-constrained devices.

## Figures and Tables

**Table 1:** Notations.

Notations	Meanings
EV	Electric vehicle
EAG	Energy aggregator
OP	Operator
$E_{p}$	Elliptic curve over a finite field, where *p* is a large prime number
*G*	Base point in $E_{p}$
$ID_{i}$/$PW_{i}$	Identity/password for entity *i*
$r_{i}$	Private key for entity *i*
$PK_{i}$	Public key for entity *i*
*a*, *b*	Random number
$k_{op}$	Random number from OP
h(·)	Hash function
‖	Concatenation operation
⊕	XOR operation
SK	Session key

**Table 2:** Notations for BAN logic.

Notation	Description
A|≡X	*A***believes** statement *X*
#X	Statement *X* is **fresh**
A⊲X	*A***sees** statement *X*
A|∼X	*A* once **said** *X*
A⇒X	*A***controls** statement *X*
$<X{>}_{Y}$	Formula *X* is **combined** with formula *Y*
${\left\{X\right\}}_{K}$	*X* is **encrypted** under key *K*
$A\overset{K}{↔}B$	*A* and *B* may use **shared key** *K* to communicate
$\overset{K}{{}_{⟶B}}$	*B* has *K* as a **public key**
SK	Session key used in the current session

**Table 3:** Computation costs for related schemes.

	Lai et al. [44]	Qiu et al. [45]	Huang et al. [20]	Proposed
**EV**	$7T_{sm-bp}+T_{pa-bp}+T_{mtp}$	$2T_{sm-ecc}+3T_{pa-ecc}+8T_{h}$	$T_{sm-ecc}+2T_{pa-ecc}+4T_{h}$	$T_{enc-ecc}+9T_{h}$
	≈16.272 ms	≈0.8902 ms	≈0.4460 ms	≈1.1709 ms
**EAG**	$2T_{bp}+5T_{sm-bp}+T_{pa-bp}+T_{mtp}$	$2T_{sm-ecc}+4T_{pa-ecc}+5T_{h}$	$2T_{sm-ecc}+2T_{pa-ecc}+7T_{h}$	$T_{dec-ecc}+4T_{h}$
	≈21.276 ms	≈0.8917 ms	≈0.8883 ms	≈0.6104 ms

**Table 4:** Communication costs.

Scheme	Total Messages	Communication Cost
**Lai et al. [44]**	2	2560 bits
**Qiu et al. [45]**	3	2656 bits
**Huang et al. [20]**	5	4288 bits
**Proposed**	4	1824 bits
